# Micrometer‐scale tPA beads amplify plasmin generation for enhanced thrombolytic therapy

**DOI:** 10.1002/btm2.70012

**Published:** 2025-03-03

**Authors:** Matthew J. Osmond, Fabrice Dabertrand, Nidia Quillinan, Enming J. Su, Daniel A. Lawrence, David W. M. Marr, Keith B. Neeves

**Affiliations:** ^1^ Department of Bioengineering University of Colorado Denver, Anschutz Medical Campus Denver Colorado USA; ^2^ Department of Anesthesiology University of Colorado Anschutz Medical Campus Denver Colorado USA; ^3^ Department of Pharmacology University of Colorado Anschutz Medical Campus Denver Colorado USA; ^4^ Department of Internal Medicine, Division of Cardiovascular Medicine University of Michigan Ann Arbor Colorado USA; ^5^ Department of Chemical and Biological Engineering Colorado School of Mines Golden Colorado USA; ^6^ Departmet of Pediatrics, Section of Hematology, Oncology, and Bone Marrow Transplant University of Colorado Anschutz Medical Campus Denver Colorado USA; ^7^ Hemophilia and Thrombosis Center University of Colorado Anschutz Medical Campus Denver Colorado USA

**Keywords:** fibrin, fibrinolysis, microparticles, plasmin, plasminogen activators, stroke, thrombolysis

## Abstract

Rapid restoration of blood flow is critical in treating acute ischemic stroke. Current thrombolytic therapies using tissue plasminogen activator (tPA) are limited by low recanalization rates and risks of off‐target bleeding. Here, we demonstrate that a remarkably simple adjustment—using micrometer‐scale rather than sub‐micrometer particles to immobilize tPA—fundamentally improves thrombolysis. By merely increasing the particle diameter from 0.1 to 1.0 μm, we achieve a dramatic shift in lysis dynamics: 1.0 μm tPA‐beads generate higher plasmin flux, readily overcome antiplasmin inhibition, and trigger a self‐propagating cascade of fibrinolysis. This leads to near‐complete clot dissolution at tPA doses nearly 100‐fold lower than standard free tPA, both in vitro and in a murine model of acute ischemic stroke. Within minutes, low‐dose 1.0 μm tPA beads fully restore blood flow, outperforming conventional therapies. Our results show that simply scaling up particle size can resolve kinetic and transport barriers in thrombolysis, offering a promising advancement in stroke treatment with potential applications in other thrombotic disorders.

**Translational Impact Statement**

A straightforward change—making drug‐carrying particles larger—enables more rapid and complete clot removal at reduced doses, offering a potentially safer, more efficient way to restore blood flow in stroke patients.

## 1 INTRODUCTION

Achieving timely recanalization of occluded cerebral arteries is central to minimizing brain injury in acute ischemic stroke. Current pharmacological therapy relies on enzymatic breakdown of the fibrin scaffold within the thrombus using recombinant tissue plasminogen activators (tPA), with alteplase and tenecteplase currently approved by the FDA. Administered within 4.5 h after symptom onset, intravenous tPA improves outcomes and is often combined with mechanical thrombectomy when feasible.^1^, ^2^ However, since the introduction of recombinant tPAs more than 30 years ago, outcomes in ischemic stroke have not changed, with only 10%–20% of large vessel occlusions recanalized with IV tPA,^3^ underscoring the need for more effective treatments.

Plasminogen activators, and tPA in particular, are and have been the strategy of choice for fibrinolysis. tPA has a high affinity (*K*_d_ = 1–2 nM) to bind to fibrin fibers; thus, it is a targeted therapy as it accumulates at the thrombus interface, enhancing local plasminogen activation.^4^, ^5^, ^6^ tPA's endogenous inhibitor, PAI‐1, circulates at a relatively low concentration (0.1–1 nM) and thus can be overcome by exogenous recombinant tPA with circulating concentrations of 10–50 nM following IV injection.^7^, ^8^ Intracerebral hemorrhage and off‐target bleeding are the most common complications with tPA therapy, occurring in 3%–8% of people receiving IV tPA for acute ischemic stroke.^9^, ^10^ In addition, systemic activation of plasminogen by tPA promotes fibrinogenolysis and activation of enzymes and inflammatory pathways that can cause tissue damage and impede wound repair.^11^, ^12^, ^13^, ^14^, ^15^

The effectiveness of tPA is hindered by kinetic and transport challenges within thrombi. tPA is most effective when bound to fibrin, which serves as a co‐factor to accelerate the activation of plasminogen to plasmin by 400‐fold.^6^, ^16^, ^17^ However, tPA and plasmin(ogen) compete for the same binding sites on fibrin fibers. This results in the counterintuitive observation that high tPA concentrations attenuate fibrinolysis due to both competition for binding sites and plasminogen depletion. Indeed, plasminogen availability is rate limiting under high tPA concentrations.^18^, ^19^, ^20^ Furthermore, the lysis pattern of tPA‐mediated thrombolysis tends to be anisotropic and incomplete; regions of high permeability within the center of the thrombus tend to lyse first, leaving small channels that partially reestablish blood flow but conversely lead to slow surface erosion of the remaining wall‐bound thrombus.^21^, ^22^

Direct infusion of plasmin, which would bypass plasminogen activation, is hampered by binding with one of its endogenous inhibitors, α2‐antiplasmin, which circulates at a high plasma concentration (~1 μM).^23^, ^24^ Local delivery of plasmin has been attempted via catheter, with success in animal models and phase I clinical trials, but has not been shown to be superior to tPA in stroke populations.^25^ Immobilization of tPA to particles, which generates plasmin locally while sidestepping the competition between tPA and plasmin for fibrin binding sites, is an alternative. This approach has been implemented primarily on sub‐micrometer scale particles, often decorated with fibrin or platelet targeting moieties or embedded with iron oxide to allow for enhanced accumulation or local hyperthermia using external magnetic fields.^9^, ^26^, ^27^, ^28^, ^29^, ^30^, ^31^ These tPA–nanoparticle constructs can increase reperfusion times in animal models of thrombosis faster than free tPA.^32^

Here, we introduce an innovative yet straightforward approach: immobilizing tPA on micrometer‐scale beads to significantly enhance local plasmin generation. Unlike previous nanoparticle‐based strategies, our method leverages the size‐dependent plasmin generation of larger beads to overcome kinetic barriers and antiplasmin inhibition, leading to rapid and efficient thrombus dissolution both in vitro and in vivo. In a murine model of acute ischemic stroke, we show that these particles entrain themselves within thrombi, lysing them from the inside out, resulting in near‐complete removal of thrombotic material in minutes. This is accomplished by using tPA doses that are almost two orders of magnitude lower than what is needed for similar recanalization times with free tPA.

## 2 RESULTS

### 2.1 Fibrinolysis with tPA beads is size dependent

To provide a faithful comparison between free and immobilized tPA on beads, we use tPA activity (mU/mL) as measured by a tPA‐specific chromogenic substrate. A standard curve was generated for tPA activity (0–2 μg/mL) and used to titrate tPA‐bead concentrations to obtain an equivalent activity per milligram of beads (Figure S1). The 0.1 μm tPA beads had approximately half the activity per mg of those of the 1.0 μm tPA beads. For all subsequent in vitro experiments, we use a tPA activity of 2.25 mU/mL for both free tPA and tPA beads, which corresponds to 1 μg/mL (14 nM) free tPA, a concentration that is comparable to the circulating concentrations of alteplase in humans following the initial bolus.^33^

**FIGURE 1:**
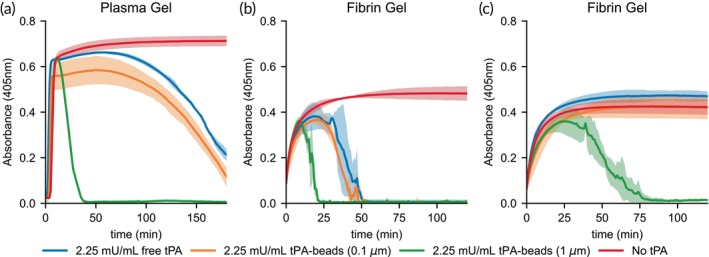
Size‐dependent fibrinolysis with tPA beads. Fibrin gels were formed with (a) normal pooled plasma, tissue factor (1.25 pM) and CaCl_2_ (8.3 mM); (b) fibrinogen (2 mg/mL), glu‐plasminogen (10 μM), thrombin (2 U/mL); (c) fibrinogen (2 mg/mL), glu‐plasminogen (10 μM), thrombin (2 U/mL), α2‐antiplasmin (1 μM) with free tPA or tPA‐beads at 37°C. Fibrinolysis was measured by absorbance (405 nm) for tPA‐beads (2.25 mU/mL) with diameters of 0.1 μm (3.55 × 10^10^ beads/mL) and 1.0 μm (1.69 × 10^8^ beads/mL) or free tPA (2.25 mU/mL). Each line and shaded region represent the average and standard deviation of *n* = 3.

For equivalent tPA activities (2.25 mU/mL), 0.1 μm tPA beads showed similar fibrinolysis rates in plasma‐derived fibrin gels as free tPA as measured by turbidity (Figure 1a) with a lysis phase that began at ~60 min and continued to the end of the experiment (180 min) while not quite reaching full lysis. Note that the 0.1 μm tPA bead does not reach the same maximum absorbance, suggesting some lysis occurred before the fibrin gel could fully form. For 1 μm tPA beads, the lysis was notably faster; there was a rapid lysis phase from 11 min to complete lysis at 38 min on average. This indicates that bead size directly influences fibrinolytic efficiency.

To determine if the differences between the 0.1 and 1 μm tPA beads were a function of plasmin inhibition by antiplasmin, the same experiment was repeated in a purified system wherein a fibrin gel was formed from fibrinogen, thrombin, and plasminogen with or without antiplasmin at its plasma concentration (1 μM) (Figure 1b,c). In the absence of antiplasmin, the trends are similar to the plasma gel; the 1 μm tPA beads have a shorter lag time and faster lysis rate compared to 0.1 μm tPA beads and free tPA. In the presence of antiplasmin, only the 1 μm tPA beads can induce fibrinolysis, albeit at a prolonged lag time and rate compared to experiments without antiplasmin. These data suggest that plasmin generation on 1 μm tPA beads can bind to fibrin fibers and overcome inhibition by antiplasmin more readily than 0.1 μm tPA beads.

### 2.2 Local plasmin flux from tPA bead surface dictates global fibrinolysis

**FIGURE 2:**
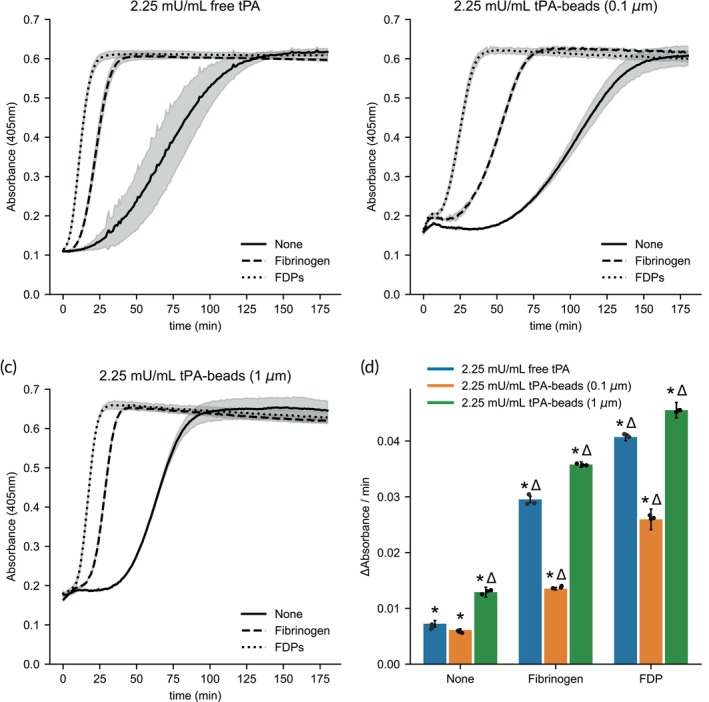
Plasmin generation of tPA beads. Plasmin generation was measured using chromogenic substrate S‐2251, glu‐plasminogen, and 2.25 mU/mL tPA activity with (a) free tPA, (b) 0.1 μm t‐PA beads, or (c) 1 μm tPA beads and its co‐factors fibrinogen or fibrin degradation product (FDP) at 37°C. Each line and shaded region represent the average and standard deviation of *n* = 3. (d) Slope of the reaction curves at 50% of the maximum absorbance for each condition. Bars and error bars represent the average and standard deviation of *n* = 3. *p < 0.001 represents the significant difference between matching tPA conditions for a given co‐factor (comparison of grouped blue, orange, and green bars). Δ*p* < 0.001 represents the significant difference between co‐factors for a given tPA condition (comparison of like colored bars).

To determine how plasmin generation on bead surfaces compared to tPA activity, cleavage of a plasmin‐specific chromogenic substrate was measured. Here, a suspension containing 2.25 mU/mL tPA activity of 0.1 and 1 μm tPA beads was compared to free 2.25 mU/mL free tPA (Figure 2). In the absence of any co‐factors, the plasmin generation, as calculated by the slope of the kinetic absorbance curve, on 1 μm tPA beads was about twofold higher than that of 0.1 μm tPA‐beads and free tPA with equivalent activity. In the presence of co‐factors of tPA, fibrinogen, and FDP, plasmin generation was significantly increased for all conditions. This observation suggests that even when the tPA is immobilized on the bead, it is still able to form a complex with these co‐factors and plasminogen to increase the catalytic rate of the enzymatic reaction. However, the increased plasmin generation with these co‐factors on 0.1 μm tPA beads was not as prominent as for free and 1 μm tPA beads.

### 2.3 Micrometer tPA‐beads catalyze self‐propagating internal fibrinolysis

**FIGURE 3:**
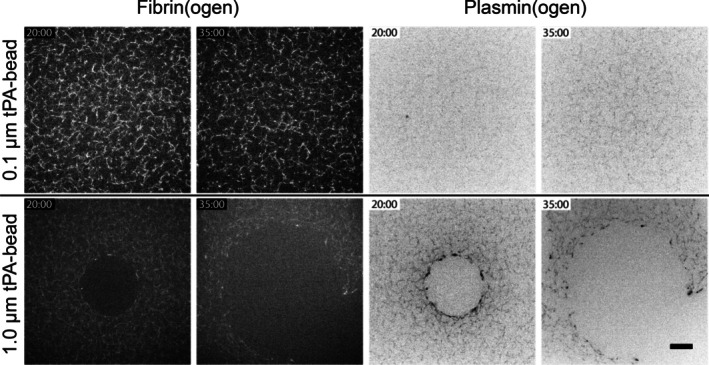
Internal fibrinolysis at the bead scale. Fluorescent micrographs of internal lysis of fibrin gels in a purified system (fibrinogen, thrombin, and plasminogen) by 0.1 μm tPA beads, or 1 μm tPA beads at 20°C. The fibrin network was labeled using 40 μg/mL Alexa Fluor 647 labeled fibrinogen (1:25 labeled:unlabeled) and Pacific blue labeled glu‐plasminogen (1:10 labeled:unlabeled). Time stamp in minutes:seconds. Scale bar = 20 μm.

To explore fibrinolysis and the length scale of the tPA beads, we observed fibrin fiber lysis around individual beads by confocal microscopy, using fluorescently labeled fibrin(ogen) and plasmin(ogen) (Figure 3). A more dilute tPA activity of 5.62 μU/mL of the 0.1 and 1 μm tPA beads was used here such that there was only a one bead per field of view. For both conditions, plasmin(ogen) appears to accumulate on fibrin fibers and spreads radially from the bead. For the 0.1 μm tPA beads, the templating occurs, but little lysis is observed over 1 h.

**FIGURE 4:**
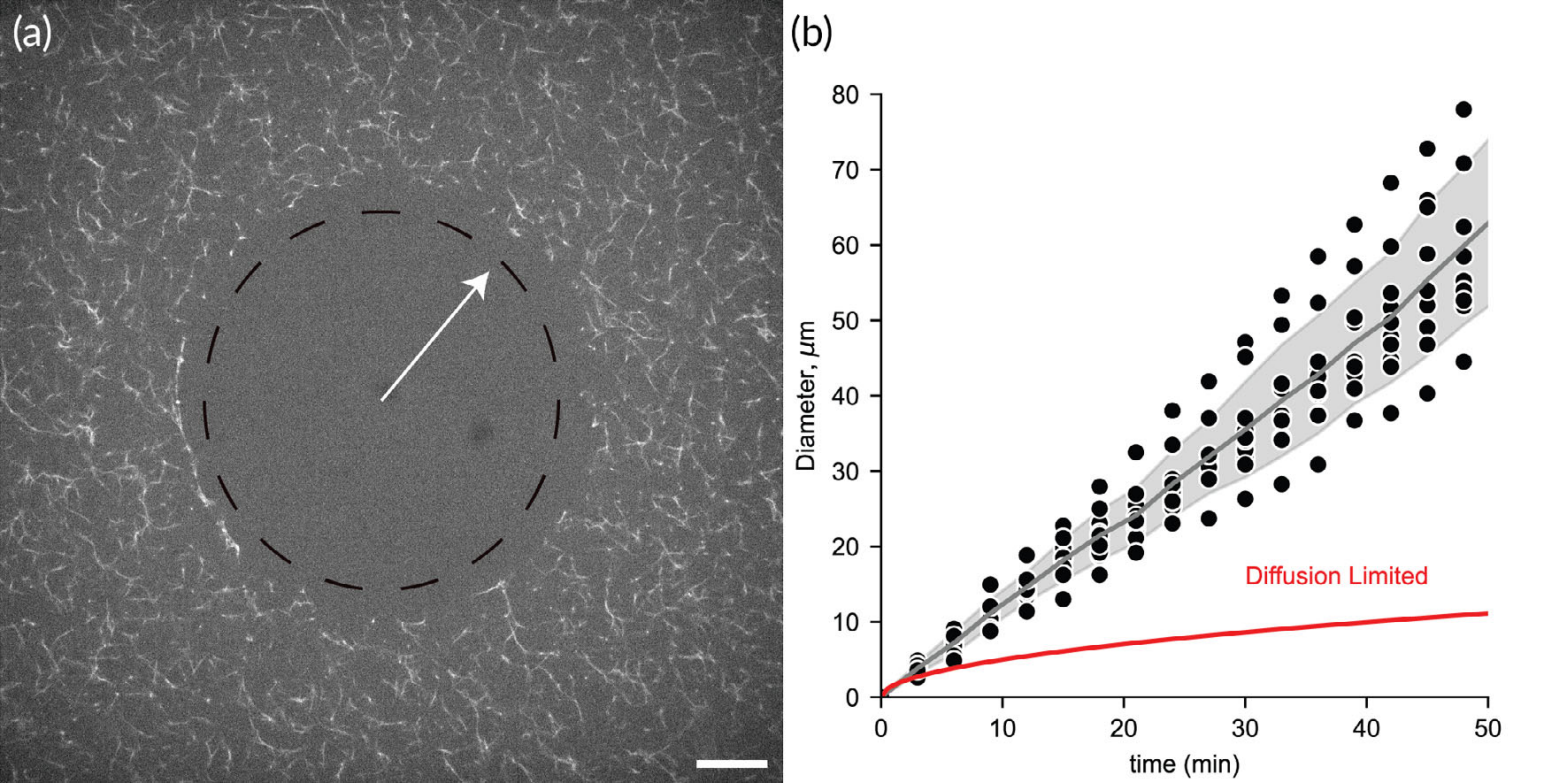
Growth rate of fibrinolytic halo around tPA beads. (a) Fluorescent micrograph illustrating the lysis front around a 1.0 μm tPA bead at *t* = 50 min. The fibrin network was labeled using 40 μg/mL Alexa Fluor 647 labeled fibrinogen (1:25 labeled:unlabeled). Scale bar = 20 μm. (b) Halo diameter as a function of time recorded from *n* = 10 different tPA beads. The line and shaded region represent the average and standard deviation. The red line represents a simple diffusion model with a diffusion coefficient that gives the same initial change in diameter as the real data.

For the 1 μm tPA beads, a halo of lysed fibrin forms around the bead, eventually propagating beyond the field of view and coalescing with halos of other nearby beads (Movie S1). The perimeter of the halo is enriched in plasmin(ogen) compared to the bulk solution. These halos, once initiated, self‐propagate over a length scale of 100 s of microns, much larger than the 1 μm beads. The diameter of these halos grows linearly with time, suggesting this is not a diffusion‐limited process that scales with the square root of time (Figure 4). That is, plasmin does not appear to be diffusing from the tPA bead to the lysis front but rather binding and unbinding to or crawling along fibrin fibers.

### 2.4 Simulation of plasmin flux and penetration

**FIGURE 5:**
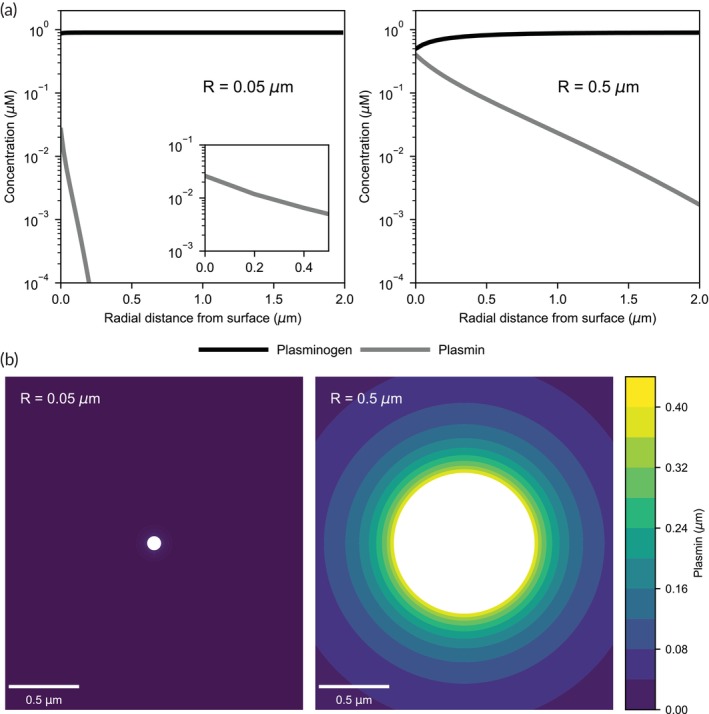
Simulation of plasmin generation on beads. A reaction‐diffusion model of plasmin generation on tPA beads is used to predict the concentration profiles (a) of plasminogen and plasmin as a function of radial distance from the bead surface in the absence of anti‐plasmin. Contour plots (b) of plasmin concentration on 0.1 and 1.0 μm tPA beads in the absence of anti‐plasmin. Reaction constants used were calculated for a temperature of 20°C. The initial concentration of plasminogen was 0.9 μM.

To aid in the interpretation of the single bead internal fibrinolysis assays (Figure 4), the plasmin flux from the surface of 0.1 and 1 μm tPA beads and its penetration distance were estimated with a reaction–diffusion model (Figure 5). In the model, plasminogen diffuses to the surface of the beads, is activated by tPA to plasmin, and plasmin diffuses away from the bead and, in the cases noted, binds to antiplasmin. The model solution is governed by two Dahmköhler numbers, which are dimensionless ratios of the reaction rate to the diffusion rate (see Supplemental Table 1). The first Dahmköhler number, Da_bead_, is the ratio of the reaction rate of plasminogen activation to plasmin by tPA to the diffusion rate of plasminogen to the bead surface. The second Dahmköhler number, Da_ap_, is the ratio of antiplasmin binding to plasmin to the diffusion rate of plasmin away from the bead surface. When the Da <1, the process is reaction‐limited; that is, the reaction is the rate‐limiting step. Conversely, when the Da >1, the process is diffusion‐limited, and the diffusive transport is rate‐limiting.

For the conditions of the single bead lysis experiments (Figure 3), there is no antiplasmin, and the Da_bead_ = 0.20 for the 0.1 μm tPA bead and Da_bead_ = 2.0 for the 1 μm tPA bead. This suggests that there is a transition in the dynamics of plasmin generation for the smaller bead, which is reaction‐limited, to the larger bead, which is diffusion‐limited. The factor of 10 difference follows the linear scaling of Da_bead_ with bead radius. This transition from reaction‐limited to diffusion‐limited is reflected in the plasminogen concentration profile from each of the two sized bead surfaces; there is no appreciable depletion of plasminogen near the 0.1 μm tPA bead, while the near‐surface plasminogen concentration is roughly half that of the bulk concentration. As the 1 μm tPA bead creates a nearly two orders of magnitude higher plasmin generation than 0.1 μm tPA beads (260 fmol/s vs. 4.6 fmol/s), the penetration of plasmin is significantly higher. For example, using 5 nM as a benchmark as this plasmin concentration lyses fibrin fibers in 10s of min^34^ similar to the time scale of our single bead lysis experiments, the model predicts this plasmin concentration 85 nm from the 0.1 μm bead and 1.9 μm radially from the 1 μm bead. Given that the pore size of fibrin or plasma gel made from 2 to 3 mg/mL of fibrinogen is on the order of 0.5–1.2 μm,^35^, ^36^ these calculations suggest that plasmin generated on 0.1 μm tPA beads is unlikely to reach fibrin fibers beyond those it is directly in contact with or within ~100 nm. Conversely, 1 μm tPA beads yield nanomolar concentrations at micrometer penetration depths likely affecting many nearby fibrin fibers. The model predicts the transition between these two regimes (Da_bead_ = 1) occurs at a particle diameter of 0.88 μm.

To extend this analysis to the turbidity assays (Figure 1b,c), we add antiplasmin into the model. In this, the Da_bead_ values are within a factor of two due to temperature differences with the single bead lysis experiments (see Supplemental Materials); however, Da_ap_ is dramatically different for the two bead sizes. Da_ap_ = 0.43 for the 0.1 μm tPA beads and Da_ap_ = 43 for the 1 μm beads. This hundred‐fold difference follows from the radius‐squared dependence of Da_ap_ and suggests that antiplasmin has little effect on plasmin penetration from smaller beads but dictates how far plasmin can penetrate from larger ones (Figure S2). This supports turbidity results showing an attenuation in lysis rate with 1 μm tPA beads in the presence of antiplasmin. However, the model does not explain why 0.1 μm tPA beads show no observable lysis.

### 2.5 Micrometer tPA beads accelerate external fibrinolysis compared to free tPA

**FIGURE 6:**
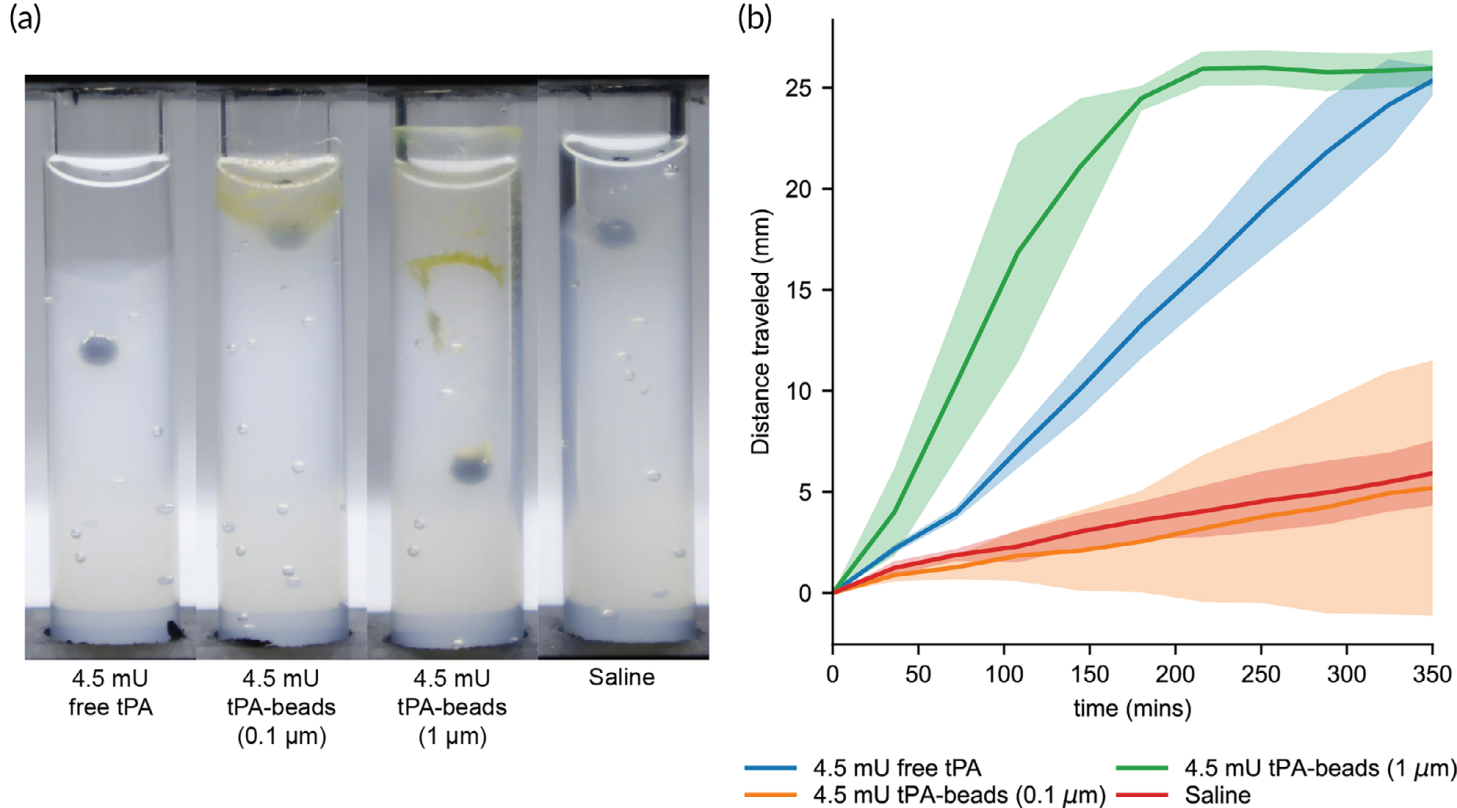
External fibrinolysis measured by the ball sedimentation assay. (a) Fibrin gels were formed using fibrinogen (2 mg/mL), plasminogen (0.2 μM), and thrombin (2 U/mL), then the addition of a 100 μL bolus of (from left to right), 0.1 μm beads, 1 μm beads, and free tPA (each containing 4.5 mU tPA activity) and a saline‐only control. The photograph shows the displacement of a 2 mm steel ball bead after 2 h of lysis at 37°C. (b) Distance traveled for stainless steel beads over time. Each line and shaded region represent the average and standard deviation of *n* = 3.

The internal fibrinolysis experiments presented in the previous section show that when beads are mixed throughout a fibrin gel, the 1 μm beads have a faster lysis rate than the same activity of 0.1 μm tPA beads and free tPA. To determine how tPA beads lyse a fibrin gel when introduced at an interface, we used a ball sedimentation assay wherein the velocity of the ball is used as a proxy for fibrinolysis rate (Figure 6).^37^, ^38^ In this, a 4.5 mU tPA bolus in 100 μL was placed on top of a fibrin gel for each condition (free tPA, 0.1 and 1 μm tPA beads). This bolus was chosen to match that used in the bolus used in the photothrombotic murine stroke model (next section). As apparent in Figure 6a, the 1 μm tPA beads have a faster ball drop rate, reaching the bottom of the cuvette within 200 min compared to the free tPA, which takes 350 min (Figure 6b). The 0.1 μm tPA beads do not significantly lyse the clot over the observation period, as the distance traveled is comparable to the saline negative control, reflecting the settling of the dense steel ball into the porous fibrin gel rather than any lysis.

### 2.6 Micrometer tPA beads rapidly reestablish pre‐occlusion blood flow in a photothrombotic stroke model

**FIGURE 7:**
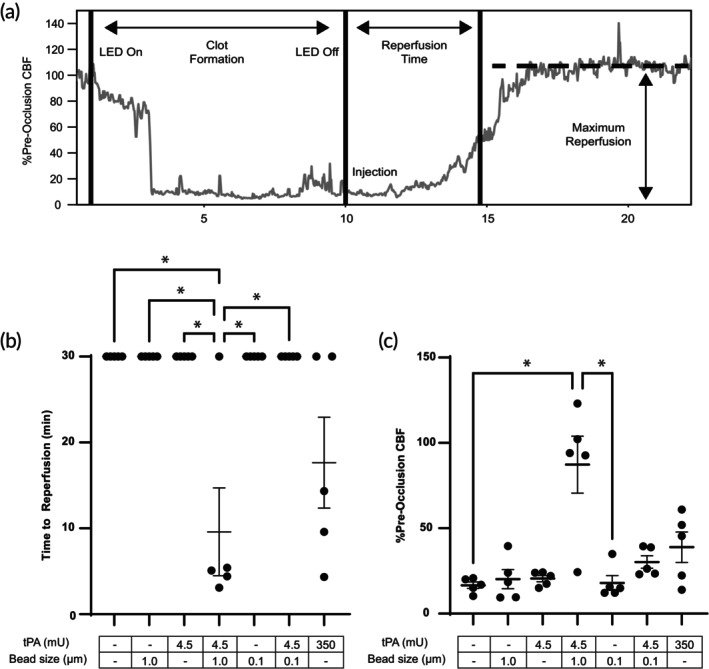
tPA‐bead‐mediated thrombolysis in the murine photothrombotic stroke model. (A) A representative laser doppler flowmeter curve generated by measuring blood flow through the middle cerebral artery of a mouse during thrombus formation and lysis for a 4.5 mU dose of 1 μm tPA beads. The time to reperfusion is defined as the time from the onset of the treatment to the inflection point where significant reperfusion occurs. The maximum perfusion is defined as the maximum achieved blood flow that happens after treatment onset. The (B) time to reperfusion and (C) maximum reperfusion for saline, 1.0 μm beads (no tPA), 4.5 mU free tPA, 4.5 mU 1.0 μm tPA beads, 0.1 μm beads (no tPA), 4.5 mU 0.1 μm tPA beads, and 350 mU free tPA. *n* = 5 per condition. *p < 0.01.

The in vitro experiments presented above show that 1 μm tPA beads induce faster internal and external fibrinolysis than comparable concentrations or doses of free tPA and 0.1 μm tPA beads and that they display a unique localized halo‐like lysis pattern. To determine if these observations translate to in vivo thrombolysis, we used a photothrombotic thrombosis model in the middle cerebral artery of the mouse. Saline, free tPA, tPA‐beads (0.1, 1 μm), and beads (nonfunctionalized, 0.1, 1 μm) were delivered as a bolus through the tail vein. A tPA dose of 4.5 mU was chosen to reflect a systemic concentration comparable to in vitro experiments (2.25 mU/mL × ~ 2 mL blood volume = 4.5 mU). We also included a 350 mU tPA dose (5 mg/kg) as this has been shown to be an effective dose for thrombolysis in this model.^39^ Blood flow was measured by laser doppler flowmeter and two metrics of thrombolysis were calculated: time to reperfusion and % pre‐occlusion cerebral blood flow (Figure 7). Only the 4.5 mU dose of the 1 μm tPA beads and the 350 mU dose of the free tPA showed measurable blood flow in the occluded vessel (Figure S3 and Figure 7b,c). In four out of the five trials with 1 μm tPA beads, the time to reperfusion was ~5 min, with one trial showing no reperfusion. In three of the five trials with 350 mU free tPA, the time to reperfusion was more variable and ranged from 5 to 15 min, with two trials showing no reperfusion. In the cases where reperfusion occurred for the 1 μm tPA beads, there was nearly complete rescue of blood flow to pre‐occlusion levels. In contrast, for the 350 mU free tPA cases where there was reperfusion, there was a 50%–60% rescue of pre‐occlusion blood flow.

**FIGURE 8:**
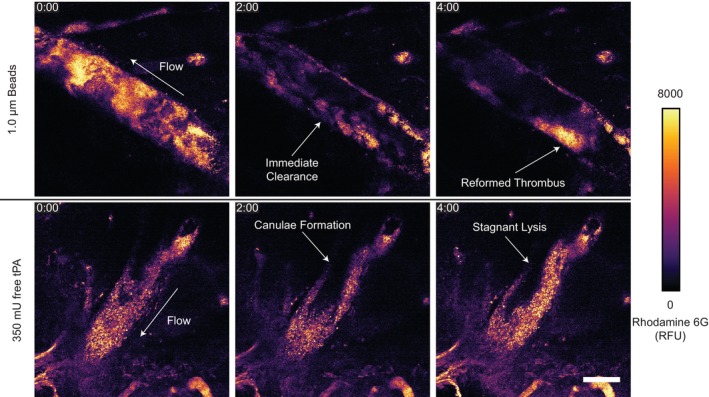
Thrombolysis observed by intravital two‐photon microscopy in the mouse middle cerebral artery. Blood cells were stained using rhodamine 6G (2.5 mg/kg) where the higher intensity portions of the heat‐mapped images indicate higher cell density within the thrombus. Treatments of 4.5 mU 1 μm tPA beads and 350 mU free tPA were injected through the tail vein. The direction of flow is illustrated with arrows in the initial frames. Scale bar = 50 μm. Time stamp is minutes:seconds.

To further explore these two conditions, intravital microscopy was used to visualize the thrombus and its dissolution (Figure 8). The lysis pattern with a 350 mU dose of free tPA was the formation of small canulae that eventually re‐canulated the vessel but, upon restoration of blood flow, showed little additional lysis of the thrombus attached to the vessel wall over the observation period of 30 min (Movie S1). The 4.5 mU dose of 1 μm tPA beads caused the entire thrombus to slough off the vessel wall and be completely removed by blood flow (Movie S2). Following the removal of the occlusive thrombus, subsequent blood cell accumulation on the vessel wall was also removed within minutes. Fluorescently labeled 1 μm beads without tPA were injected and found to entrain themselves throughout the thrombus, suggesting that tPA beads were able to induce lysis from the inside out (Figure S4).

## 3 DISCUSSION

Our study demonstrates that immobilizing tPA on micrometer‐scale beads significantly enhances local plasmin generation compared to smaller beads and free tPA, leading to rapid and efficient fibrinolysis both in vitro and in vivo. The larger bead size is critical, as it supports plasmin generation that overcomes transport limitations and facilitates a self‐propagating wave of fibrinolysis. These same tPA beads can rapidly re‐establish blood flow in a murine model of ischemic stroke at a dose almost two orders of magnitude less than that required for free tPA. The immobilization of tPA to a bead is fundamentally different from the mechanism by which free tPA generates plasmin. In the case of free tPA, a complex between tPA‐fibrin‐plasminogen localizes plasmin generation to the fibrin fiber and minimizes its inhibition by antiplasmin. In the case of immobilized tPA, a similar complex can form with fibrinogen or fibrinogen degradation products to serve as a co‐factor; however, the plasmin generated on the bead surface must be transported to fibrin fibers. The implications of these differences are discussed below.

By standardizing tPA activity across our experiments, we can directly compare the effects of bead size on plasmin generation. Our results show that 1 μm tPA‐beads generate significantly more plasmin than both 0.1 μm beads and free tPA, highlighting the importance of bead size in enhancing fibrinolytic activity. An approximately 200‐fold higher particle density of 0.1 μm beads is needed to match the tPA activity of 1 μm beads, which is twice as many as expected based on surface area. This suggests a difference in tPA enzymatic activity on the two sized beads, as supported by observations that, at equivalent tPA activities, 1 μm beads have higher plasmin generation than 0.1 μm beads, with a profound difference in tPA co‐factors, fibrinogen, and fibrin degradation products. Potential explanations for these observations include differences in curvature and steric limitations for substrate and co‐factor binding. A reaction–diffusion model of plasmin generation on tPA beads, even with comparable reaction kinetics between the two sizes, predicts that plasmin generation is reaction limited on the smaller beads in agreement with our experimental observations.

Comparing free tPA and 1 μm tPA beads shows significantly faster and different lysis patterns in vitro. Plasmin generation with co‐factors for a given tPA activity is slightly higher, 10%–15%, for 1 μm tPA beads compared to free tPA. Yet, the rate of internal fibrinolysis, as measured by turbidity, and external fibrinolysis, as measured by the ball sedimentation assay, is markedly faster for 1 μm tPA beads. Examining fibrinolysis at the bead scale suggests that this marked difference is due to a self‐propagating fibrinolysis front that emanates away from the beads as a halo decorated with a high concentration of plasmin(ogen). These halos grow linearly with time to the size of hundreds of micrometers, much faster than a diffusion‐mediated process. One potential explanation is the bundling of fibrin fibers upon lysis; fibrin fibers have an inherent tension and, when cut, retract like a taut rope, forming bundles of fibers.^40^, ^41^, ^42^, ^43^ The thickening band of fluorescence in the growing halos that form around tPA beads could be indicative of such fiber bundling. The proximity of fibers in a bundle could allow for plasmin to unbind and rebind between fibers at a favorable rate compared to diffusion or inhibition by antiplasmin or, alternatively, enhance the rate of crawling between binding sites.^16^, ^44^

At a 77‐fold lower dose, 1 μm tPA beads result in faster and more complete lysis than free tPA. This remarkable difference likely reflects the high plasmin generation on the bead surface that both overcomes antiplasmin inhibition and appears to bind to adjacent fibrin fibers at high concentrations in vitro. The thrombi formed in the photothrombotic model used in this study are porous enough to allow for interstitial plasma flow as evidenced by the entrainment of 1 μm particles through the thrombi. This could explain why in vivo lysis is much faster than in vitro lysis; the nonzero interstitial velocity through the thrombus replenishes plasminogen near the bead surface, which our calculations suggest is depleted in a purely diffusive environment. The thrombi were only 10 min old before treatment was introduced and, as such, they were fibrin‐rich, porous, and potentially not highly contracted by platelets. Older clots that are more cellular and retracted will likely be harder to lyse; however, we have previously shown in vitro and in zebrafish models of thrombosis that tPA immobilized to superparamagnetic beads and assembled into wheel‐like microbots can be directed to and driven into platelet‐rich thrombi.^45^, ^46^ The pattern of lysis was different between free tPA and tPA beads. Free tPA initiates small channels that slowly widen at the blood‐thrombus interface, indicative of more permeable regions in the middle of the clot.^21^, ^47^, ^48^ tPA beads, however, lyse the clot from the inside out, resulting in failure‐like events where the entire thrombus loses mechanical fidelity and dislodges from the vessel wall.

Our reaction–diffusion model predicts that a transition from a reaction‐limited regime to a diffusion‐limited regime occurs at a particle diameter of 0.88 μm, slightly smaller than the 1 μm beads used in our experiments. Most studies of drug delivery systems of surface‐immobilized tPA consist of particles that range in diameter from 10 to 100 nm, and thus likely sit within the reaction‐limited regime.^32^ The synthesis of many of these approaches, including polymer and liposomes, is amenable to scaling to larger particles. An important caveat is that while the rate of plasmin generation is particle size‐dependent, it is also dependent on the surface density of tPA on the particle. Particle roughness, surface chemistry, and linkage chemistry between the particle surface and the tPA influence its surface density, a parameter that is hard to measure and rarely reported. There have been studies of larger, micrometer‐scale tPA delivery systems, most notably immobilized tPA on red blood cells (RBC), whose 8.5 μm diameter and 1.2 μm thickness would be well within the diffusion‐limited regime.^49^ tPA‐RBC in a murine pulmonary emboli model can lyse 80% of microemboli compared to 20% of free tPA. However, in a murine carotid artery thrombosis, only 30% of pre‐occlusion blood flow is recovered, likely due to the inability of large tPA‐RBC to penetrate a preformed clot. Here, our 1 μm particles were able to penetrate newly formed clot in a photothrombotic model; however, older clots that have retracted and may have more cellular content could be more challenging to lyse. Thus, engineering strategies that balance clot penetration with size‐enhanced plasmin generation are vital for optimizing in vivo efficacy. Alternatively, external electromagnetic or acoustic fields could aid in the penetration of larger micrometer‐scale particles.^20^, ^45^, ^50^

Intravital microscopy of clot dissolution with 1 μm beads shows rapid lysis, but the resultant emboli could occlude downstream vessels of the brain and lungs. It is possible that bead‐clot structures will continue to lyse as they propagate downstream, but that remains to be measured as measures of the distribution of clots and beads following lysis are a limitation of this study. Studies of similarly sized particles indicate sequestration into organs of the reticuloendothelial system, including the liver, lung, and spleen.^51^ Specific biodistribution and clearance mechanisms of tPA beads are important next steps needed to evaluate the translational potential of this approach.

In summary, this study shows that immobilized tPA‐mediated plasmin generation at the micrometer scale supports rapid fibrinolysis in vitro and in vivo. It does so by overcoming mass transfer barriers, inhibition by antiplasmin, and populating fibrin fibers with a plasmin concentration that can sustain a self‐propagating wave of fibrinolysis. As such, the lysis patterns are fundamentally different from conventional tPA therapies where plasmin and tPA are co‐localizing but also competing for binding sites on fibrin fibers. By achieving rapid recanalization at significantly lower tPA doses, this approach has the potential to minimize the risk of hemorrhagic complications associated with high‐dose tPA therapy. The ability to efficiently dissolve clots with lower drug amounts could improve safety profiles and expand the therapeutic window for patients with acute ischemic stroke.

## 4 MATERIALS AND METHODS

### 4.1 Materials

Dynabeads® MyOne™ Carboxylic Acid (cat# 65011) and Fluospheres™ Carboxylate‐modified microspheres 1.0 μm diameter (cat# F8823), 0.1 μm diameter (cat# F8803), and 96‐well optical bottom plates (cat# 265301) were obtained from Thermo Fisher Scientific (Waltham, MA). 1‐ethyl‐3‐[3‐dimethylaminopropyl] carbodiimide hydrochloride (EDC) (cat# c1100‐100 mg) and N‐hydroxysulfosuccinimide (Sulfo‐NHS) (cat# c1102‐1gm) were obtained from Proteochem (Hurricane, UT). Recombinant human tissue plasminogen activator, Cathflo® Activase® (tPA, alteplase, Genentech, San Francisco, CA) was obtained from the Hemophilia and Thrombosis Center pharmacy. Chromogenic substrate S‐2288 (cat# S820852) was obtained from Diapharma (West Chester, OH). Purified human lys‐plasmin (cat# IHUPLMLYS1MG) and plasmin chromogenic substrate (cat# IAFPLMCGSLY25MG) were obtained from Innovative Research (Novi, MI). Pooled normal plasma (cat# 0010) was obtained from George King Bio‐Medical Inc. 8 × 40 mm clear shell vials (cat# 50‐978‐400) were obtained from FisherScientific (Watham, MA). Bovine serum albumin (BSA) (cat# A2153‐100G), Rose Bengal (cat# 330000), bovine thrombin (cat# T4648), 2‐(N‐Morpholino) ethanesulfonic acid hydrate (MES) (cat# m8250), HEPES (cat# H3375), and sodium dodecyl sulfate (SDS) (cat# 7910) were obtained from Sigma Aldrich (St. Louis, MO). Human fibrinogen depleted of plasminogen, von Willebrand Factor, and fibronectin (FIB3), human glu‐plasminogen (HPG 2001), and α2‐antiplasmin (α2AP) were obtained from Enzyme Research Laboratories (South Bend, IN). Dade Innovin (tissue factor) was obtained from Siemens. HEPES buffered saline (HBS) was made with 20 mM HEPES and 100 mM sodium chloride pH 7.5. HEPES buffer used for purifying tPA was made with 0.3 M HEPES at pH 7.4. Coupling buffer was made from 50 mM MES and 0.01% SDS at pH 6.5.

### 4.2 Synthesis and characterization of tPA beads

The lyophilized alteplase was dissolved in HEPES buffer (1 mg/mL) and transferred to a slide‐a‐lyzer (MWCO 10,000) (Pierce, cat# 66455) dialysis cassette and dialyzed in 500 mL HEPES buffer at 4°C for 4 h. This is necessary to remove arginine from the Cathflo® formulation, which can interfere with the linkage chemistry. After dialysis, the solution was removed from the cassette and the concentration measured using spectrophotometry (NanoDrop One, Thermofisher) and diluted to 1 mg/mL in HEPES buffer. The solution was stored at −80°C. Both 0.1 and 1 μm beads were functionalized in 1 mg batches. Beads were added to microcentrifuge tubes and washed by suspending in coupling buffer (50 mM MES buffer, 0.01% SDS, pH 6.5) centrifuging at 3000*g* for 5 min, and removing the supernatant. Next, EDC (100 μL, 40 mg/mL) and sulfo‐NHS (100 μL, 40 mg/mL), both in coupling buffer, were added to the beads and mixed on a rotator for 15 min. The beads were then washed with coupling buffer to remove excess EDC/NHS. The dialyzed tPA (30 μg in 200 μL coupling buffer) was added to the bead suspension and gently inverted until the beads were fully dispersed. The beads were then mixed on a rotator at 4°C. After reacting for 4 h, the beads were washed once with coupling buffer and blocked using 2% BSA in 0.9% saline for 5 min, washed three times to remove any excess tPA or BSA, and stored in 0.9% saline. All beads were either used immediately or frozen at −80°C. All steps outlined above were conducted using sterile solutions in a sterile environment.

### 4.3 tPA activity measurement with colorimetric substrate

Tris buffer (50 μL, pH 8.4) and 50 μL of the free tPA (0–2 μg/mL final) or tPA beads (diluted 100x, ~10 μg beads/mL final) were added to each well of a 96‐well optical bottom plate and mixed by pipetting up and down. Next, the tPA colorimetric substrate S‐2288 (50 μL, 2.5 mM final, *K*_m_ = 0.3–1 mM; kcat = 26–28 s^−1^) was added to each well and the absorbance was measured at 405 nm every min for 30 min. The activity defined the slope of the absorbance versus time curve (abs/min) over 20 min and multiplied by 313 to get the activity in units of U/L according to the manufacturer's instructions.

### 4.4 Fibrinolysis turbidity assay

Normal pooled plasma (50 μL), tissue factor (Dade Innovin, 50 μL, 1.25 pM final), and CaCl_2_ (50 μL, 8.3 mM final) and free or tPA (50 μL; 2.25, 45 U/mL final) or tPA beads (50 μL, 2.25 U/mL final) in HEPES‐buffered saline (HBS) were added to a well of a 96‐well plate, and absorbance was measured at 450 nm at 37°C. Alternatively, to determine the effect of anti‐plasmin presence, a measurement was made using a purified system of fibrinogen (40 μL, 1 mg/mL final), glu‐plasminogen (40 μL, 1 μM final), thrombin (40 μL, 2 U/mL final), anti‐plasmin (40 μL, 1 μM final), and tPA or tPA beads (40 μL).

### 4.5 Formation of fibrin degradation products

Fibrin degradation products (FDP) were generated with fibrinogen (2 mg/mL), human alpha‐thrombin (2 U/mL), and Lys‐plasmin (1 μg/mL) for 16 h at 37°C, followed by the addition of Pefabloc (0.05 mM final). The solution mixture was centrifuged at 6000*g* to remove any insoluble products.^52^ The FDPs were diluted using HBS until the absorbance at 280 nm was 1.6, corresponding to a concentration of 1 mg/mL.

### 4.6 Plasmin generation assay

Reaction mixtures containing human glu‐plasminogen (10 μL, 1 μM final), plasmin chromogenic substrate (20 μL, 0.2 mM final), and tPA (20 μL, 2.25, 45 mU/mL final) or tPA beads (20 μL, 2.25 mU/mL final; 0.1, 1 μm diameter) were placed in a 96‐well assay plate. Fibrinogen (50 μL, 0.5 mg/mL final) or FDPs (50 μL, 0.5 mg/mL final) were added as co‐factors in some wells. The absorbance at 405 nm was measured every minute for 3 h.

### 4.7 Confocal microscopy of single bead fibrin lysis

Fibrinogen (1 mg/mL final), AlexaFluor 647‐tagged fibrinogen (40 μg/mL final), 0.1 μm and 1.0 μm tPA beads (0.5 μg/mL final) or free tPA (0.4 nM final) in 50 μL were mixed and followed by the addition of thrombin (2 U/mL final), glu‐plasminogen (0.9 μM final), and Pacific Blue‐tagged glu‐plasminogen (0.1 μM final) in separate 50 μL, mixed by pipetting five times and placed on a cover slip. After mixing, the time was noted and imaging started as soon as fibrin was visible and in focus. Fibrin fibers and fluorescent beads were observed using a confocal microscope (Olympus IX83 [60× objective, NA = 1.35, oil immersion]; Yokogawa CSU‐W1 spinning disk confocal unit; Thorlabs laser lines—405, 488, and 640 nm; and Hamamatsu ORCA‐Flash4.0 Digital CMOS camera) and recorded every 10 s until the fibrin network was lysed.

### 4.8 Reaction–diffusion model of plasmin generation

The spatial–temporal evolution of plasmin formed on tPA beads was modeled as a three‐component reaction–diffusion system including plasminogen, plasmin, and antiplasmin using three partial differential equations (see Supplemental Materials). These differential equations and their initial and boundary conditions were nondimensionalized and solved using the method of lines with custom Python scripts. Two Dahmköhler numbers dictate the concentration profile of plasmin: Da_bead_, which describes the rate of tPA‐mediated activation of plasminogen on the bead surface relative to the rate of plasminogen diffusion to the surface, and Da_antiplasmin_, which describes the rate of plasmin‐antiplasmin binding compared to the rate of plasmin diffusion away from the surface.

### 4.9 Ball sedimentation assay

Fibrinogen (2 mg/mL final), plasminogen (0.2 μM final), and thrombin (2 U/mL final) in a total of 1 mL of HBS were added to an 8 mm diameter × 40 mm long vial and allowed to gel for 5 min at 37°C before a 2 mm steel bead was placed on the top of the clot. The lysis was initiated by the addition of tPA or tPA beads (100 μL) to the top surface of the fibrin gel for the following conditions: 4.5 mU tPA on 0.1 μm or 1.0 μm beads, or 4.5 mU of free tPA and HBS as a control. The vials were kept at 37°C, and a time lapse image was recorded every min for 6 h using a Canon EOS M200 camera. The time it took for the ball to fall to the bottom and the distance traveled over time were measured and reported.

### 4.10 Murine photothrombotic stroke model

All procedures involving animals were reviewed and approved by the Institutional Animal Care and Use Committee of the University of Colorado (IACUC protocol number: 221). Before anesthesia, mice were warmed to dilate the tail vein, and a temporary catheter was placed for later intravenous injections. Male C57BL/6J mice (age 10–12 weeks) were deeply anesthetized using 3%–5% isoflurane and maintained using 1%–3% isoflurane. Mouse body temperature was maintained at 37°C. Animals were secured to a flat plate and visualized using a dissecting microscope (Leica M60). The left middle cerebral artery (MCA) was exposed using a process described previously.^53^, ^54^ The left temporal muscle was transected to expose the skull. The proximal branch of the MCA was located, and a 1.8 mm portion of the skull directly above the vessel was removed using a trephine (Fine science tools #18004‐18). A bare fiber optic cable (200 μm diameter) connected to a 530 nm LED (Thorlabs M530F2) was positioned over the MCA vessel, and a laser doppler flowmeter (LDF, MoorLabs DRT4) probe was positioned downstream to measure the relative blood flow. Blood flow measurements were collected for the entirety of the experiment. Each mouse was injected with Rose Bengal (40 mg/kg) and bovine thrombin (80 U/kg) in 100 μL of saline.^39^ Immediately after injection, the MCA was illuminated at full power, and the occlusion was confirmed by a drop in the flow measured by the LDF. The MCA was illuminated for 10 min and monitored to verify the stability of the clot. Treatment of the clots started ~10 min after the beginning of the illumination. A bolus (~120 μL) of saline, free tPA (4.5, 350 mU tPA), tPA beads (4.5 mU tPA, diameter 0.1, 1 μm), or unfunctionalized beads (diameter 0.1, 1 μm) were delivered using the catheter, and the LDF was monitored for 30 min to measure blood flow. Each condition was repeated with five mice.

### 4.11 Intravital microscopy of thrombolysis

The same procedures for the murine photothrombotic stroke model were used for intravital microscopy studies with some modification: To create a cranial window, the distal branch of the MCA was traced to the top of the head, and a portion of the skull was removed using a trephine. An acrylic head plate with a viewing hole was centered and securely glued around the cranial window. The window was covered with sterile saline to prevent it from drying out. The mouse was then transported to the microscope and secured using a custom stereotaxic holder for the head plate. Mice were maintained at 37°C for the entire procedure. Isoflurane was supplemented with 50 mg/kg alpha‐chloralose and 750 mg/kg urethane via peritoneal injection to provide prolonged anesthesia for the entirety of the imaging process. In addition to Rose Bengal and bovine thrombin, rhodamine 6 g (2.5 mg/kg) was co‐injected to visualize blood cells within the thrombus. The vessel was illuminated for 10 min using the 530 nm fiber optic LED to produce a thrombus, and then the mouse was immediately positioned for viewing with a Bruker Ultima Investigator multiphoton microscope using Prairie View software and a Spectra‐Physics Mai Tai® eHP DeepSee™ ultrafast laser. The system integrates two imaging channels with high sensitivity GaASP photomultiplier tubes. Excitation was done at 810 nm, and green and red fluorescence emissions were collected through 525/70 nm and 595/50 nm bandpass filters. The formed thrombus was located and recorded to ensure complete occlusion of the vessel. After the formation of the thrombus, saline, free tPA, or tPA beads were injected at the same doses described above, and the lysis was recorded for 20 min. In some experiments, nonfunctionalized beads (0.1, 1 μm) labeled with FITC of the same size were added to visualize their distribution in and around the thrombus. Each condition was repeated with three mice.

### 4.12 Statistical analysis

Results measured for significant differences were tested using the Shapiro–Wilk normality test. All data presented here was determined to have a normal distribution. A two‐way ANOVA was used to identify significant differences between the slopes of plasmin generation. Multiple comparisons were made between conditions using the same template or the same tPA source using Fisher's least significant difference method.

## AUTHOR CONTRIBUTIONS

**Matthew J. Osmond:** Investigation; writing – original draft; methodology; validation; visualization; writing – review and editing; formal analysis; data curation. **Fabrice Dabertrand:** Resources; writing – review and editing. **Nidia Quillinan:** Resources; writing – review and editing. **Enming J. Su:** Resources; writing – review and editing. **Daniel A. Lawrence:** Resources; writing – review and editing. **David W. M. Marr:** Conceptualization; methodology; validation; resources; visualization; supervision; writing – review and editing; writing – original draft; project administration; funding acquisition. **Keith B. Neeves:** Conceptualization; methodology; software; supervision; funding acquisition; resources; project administration; writing – review and editing; writing – original draft; formal analysis; visualization; data curation.

## FUNDING INFORMATION

National Institutes of Health grants R01NS102465 to K. B. N and D. W. M. M.; R01GM123746‐02S1 (multiphoton microscope); RF1NS140137, RF1NS129022, R01HL136636, and the Leducq Foundation for Cardiovascular Research (Leducq Transatlantic Network of Excellence 22CVD01 BRENDA) to F. D.

## CONFLICT OF INTEREST STATEMENT

All other authors declare they have no competing interests.

## Supporting information


**Data S1.** Supporting Information.

**Movie S1.** Growth of fibrinolytic halo around tPA beads. Confocal microscopy of the lysis front around a 1.0 μm tPA bead. The fibrin network was labeled using 40 μg/mL Alexa Fluor 647 labeled fibrinogen (1:25 labeled:unlabeled). Scale bar = 20 μm. Time stamp is hours:minutes:seconds.

**Movie S2.** Incomplete recanalization of the mouse middle cerebral artery using free tPA. Blood cells were stained using rhodamine 6 g (2.5 mg/kg) where the higher intensity portions of the heat mapped images indicate higher cell density within the thrombus. A bolus of 350 mU free tPA was injected through the tail vein. Formation of canulae is visible through the center of the thrombus. Full recanalization never occurs. Scale bar = 50 μm. Time stamp is minutes:seconds.

**Movie S3.** Spontaneous recanalization of the mouse middle cerebral artery using 1.0 μm beads. Blood cells were stained using rhodamine 6 g (2.5 mg/kg) where the higher intensity portions of the heat mapped images indicate higher cell density within the thrombus. A bolus of 1.0 μm beads with 4.5 mU of attached tPA was injected through the tail vein. Initially, some beads collected at the bottom right are visible. As time goes on, a spontaneous generation of plasmin is apparent which causes an immediate sloughing of the entire thrombus. Scale bar = 50 μm. Time stamp is minutes:seconds.

## Data Availability

The data that support the findings of this study are available from the corresponding author upon reasonable request.
